# Supplementary material for: Crystallinity Dependence of PLLA Hydrophilic Modification during Alkali Hydrolysis
Source: Polymers (Basel). 2022 Dec 25;15(1):75. doi: 10.3390/polym15010075 (PMC9823826; doi:10.3390/polym15010075)
Supplement: Supplementary file 1 [file polymers-15-00075-s001.zip › polymers-2089377-supplementary.pdf]

Supporting information for

Crystallinity Dependence of PLLA Hydrophilic Modification during Alkali Hydrolysis

Jiahui Shi [1], Jiachen Zhang [1], Yan Zhang [1], Liang Zhang [1], Yongbiao Yang [2]*, Ofer Manor [3] and Jichun You [1]*

1 Key Laboratory of Organosilicon Chemistry and Material Technology, Ministry of Education, College of Material, Chemistry and Chemical Engineering, Hangzhou Normal University, Hangzhou 311121, China

2 School of Chemistry and Chemical Engineering, Liaoning Normal University, Dalian 116029, China

3 The Wolfson Department of Chemical Engineering, Technion-Israel Institute of Technology, Haifa 32000, Israel

Corresponding author: Ass. Prof. Yang (yongbiao@lnnu.edu.cn) and Prof. You (you@hznu.edu.cn)

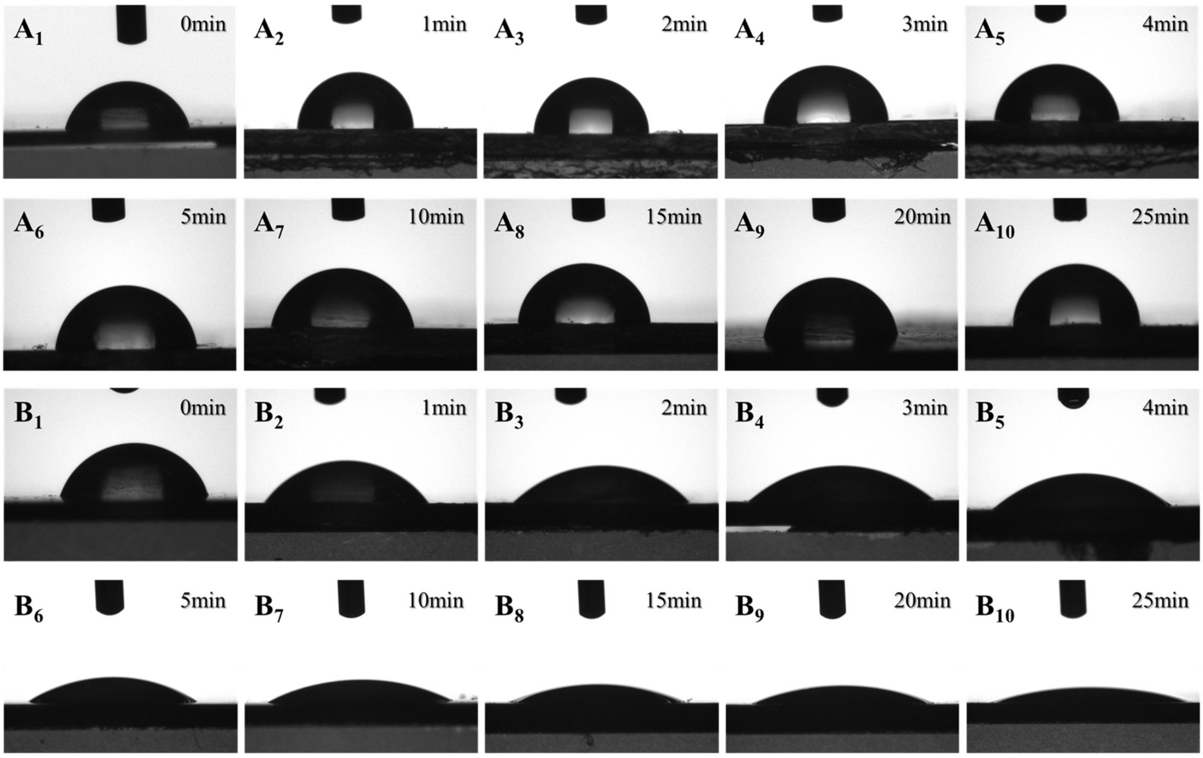

Figure S1. Contact angle pictures of modified PLLA film: amorphous PLLA films with different hydrolysis time(A_1-A_{10}); Different hydrolysis time of PLLA films annealed for 60 min (B_1-B_{10}).
